# Supplementary material for: Sex differences in body composition but not neuromuscular function following long-term, doxycycline-induced reduction in circulating levels of myostatin in mice
Source: PLoS One. 2019 Nov 21;14(11):e0225283. doi: 10.1371/journal.pone.0225283 (PMC6872155; doi:10.1371/journal.pone.0225283)
Supplement: S1 Table — Values are mean ± SEM of 10 male and 12 female control, and 11 male and 14 female DOX-treated mice. MUNE (motor unit number estimation) values are an estimate of the number of motor units in the right triceps surae muscle. SMUP (single motor unit potential size) values are the average size of motor units in the right triceps surae muscle, quantified as peak-to-peak distance in μV. μV, microvolts; g, grams. (DOCX) [file pone.0225283.s001.docx]

| Age (months) |  | 12 | 15 | 18 | 21 | 24 |
| --- | --- | --- | --- | --- | --- | --- |
| Weekly Food Consumption (g) | Female Treated  Female Control  Male Treated  Male Control | 23.7 (1.9)  24.6 (1.2)  36.4 (2.1)  30.4 (1.6) | 26.8 (0.9)  26.8 (0.6)  34.8 (0.5)  30.5 (2.4) | 27.6 (1.1)  29.3 (1.2)  36.5 (0.4)  31.9 (2.9) | 28.2 (0.8)  27.9 (1.4)  36.4 (1.2)  32.5 (2.9) | 30.0 (1.1)  30.8 (1.5)  37.0 (1.0)  32.0 (2.4) |
| Total Mass (g) | Female Treated  Female Control  Male Treated  Male Control | 34.4 (1.4)  31.0 (1.3)  37.7 (1.0)  39.0 (1.5) | 37.5 (1.6)  32.8 (1.4)  39.6 (1.2)  40.8 (1.6) | 40.5 (2.0)  35.5 (1.8)  40.9 (1.5)  42.5 (1.9) | 39.9 (2.0)  34.0 (1.8)  41.3 (1.7)  42.0 (2.0) | 38.7 (1.5)  34.4 (1.9)  40.9 (1.7)  41.5 (1.9) |
| Lean Mass (g) | Female Treated  Female Control  Male Treated  Male Control | 22.4 (0.5)  22.0 (0.6)  27.8 (0.6)  28.2 (0.7) | 24.1 (0.6)  22.6 (0.6)  29.5 (0.7)  28.6 (0.5) | 25.7 (0.6)  23.9 (0.7)  30.1 (0.8)  29.1 (0.7) | 25.3 (0.6)  23.3 (0.7)  30.1 (0.8)  29.1 (0.7) | 24.9 (0.5)  22.7 (0.7)  29.3 (0.6)  28.9 (0.6) |
| Fat Mass (g) | Female Treated  Female Control  Male Treated  Male Control | 7.2 (0.9)  4.8 (0.7)  4.6 (0.6)  5.3 (0.9) | 8.0 (1.1)  5.4 (0.7)  4.3 (0.7)  6.5 (0.9) | 9.7 (1.3)  7.2 (1.0)  4.9 (0.9)  7.6 (1.1) | 9.1 (1.3)  5.9 (0.9)  5.0 (1.0)  7.1 (1.2) | 7.6 (1.1)  6.2 (1.0)  4.3 (1.0)  6.4 (1.1) |
| MUNE (estimated #) | Female Treated  Female Control  Male Treated  Male Control | 339 (10)  354 (15)  380 (22)  332 (15) | 363 (24)  365 (23)  331 (21)  318 (19) | -  -  -  - | 218 (11)  265 (25)  278 (21)  254 (25) | 235 (20)  249 (18)  252 (18)  249 (23) |
| SMUP (µV) | Female Treated  Female Control  Male Treated  Male Control | 232 (9)  234 (18)  204 (15)  230 (17) | 206 (14)  213 (13)  247 (18)  231 (21) | -  -  -  - | 269 (11)  272 (20)  280 (25)  317 (37) | 309 (19)  328 (20)  304 (25)  314 (35) |
| CMAP (mV) | Female Treated  Female Control  Male Treated  Male Control | 78.5 (3.5)  81.8 (5.6)  74.8 (3.6)  75.4 (4.5) | 73.2 (5.0)  76.7 (5.1)  78.8 (3.3)  71.9 (5.9) | -  -  -  - | 58.3 (3.3)  68.3 (4.2)  75.3 (6.4)  75.1 (5.1) | 70.5 (4.8)  78.8 (3.3)  73.1 (2.9)  73.5 (4.3) |
