# Supplementary material for: Sex differences in body composition but not neuromuscular function following long-term, doxycycline-induced reduction in circulating levels of myostatin in mice
Source: PLoS One. 2019 Nov 21;14(11):e0225283. doi: 10.1371/journal.pone.0225283 (PMC6872155; doi:10.1371/journal.pone.0225283)
Supplement: Attachment — Submitted filename: Response to Reviewers.docx [file pone.0225283.s002.docx]

Response to Reviewers

We have now addressed those comments, as follows.

1. Reviewer Comment: For the non-expert reader please provide a more detailed description of how

motor unit number was derived from EMG data. Similarly explain how average motor unit size was

estimated.

Author Response: We have included more information in the Material and Methods section regarding

how MUNE and motor unit size is derived from the EMG measurements. We also address this comment in

the Limitations section.

2. Reviewer Comment: The results show % changes but not your estimate of the absolute number of

motor units in the muscle. It would be helpful to provide your estimates of the number of motor units in

the muscle to aid comparison with current published estimates (if any) and/or as a baseline estimate for

future studies.

Author Response: Absolute values for MUNE are included in Supplementary Table 1.

3. Reviewer Comment: Some discussion of the limitations of the MUNE method in mice (e.g. what is

known about error arising from repeated MUNE estimates for the same muscle in the same animal) and

the degree to which this might limit your ability to discern potential treatment effects.

Author Response: We have included a paragraph in the Limitations section that addresses the

assumptions of MUNE and how that might affect our results.

4. Reviewer Comment: Consider adding references to published data on motoneuron loss in old age and

discuss briefly how these might (or might not) correlate with your data on reduction in motor unit

number.

Author Response: We have added references that demonstrate similarities in motor unit loss with age that

are similar to our results (Discussion, second paragraph).

5. Reviewer Comment: Serum myostatin levels were reduced by 37% in the DOX treated animals. Given

the muscle knockdown which tissues are producing the remaining 63% of blood myostatin (or is the

muscle knockdown incomplete?), how does the blood myostatin level compare to that of preceding

studies. Do we know that the 37% reduction is biologically meaningful and if so what is the reason to

believe this? Add discussion to clarify.

Author Response: There are many tissues that express myostatin mRNA and thus are potential producers

of the protein, but in particular the tongue has high levels of expression. However, the 37% reduction is a

comparison with the levels in the untreated mice. We include in the Limitations Section a discussion of

what this reduction means, acknowledging that the Cre construct appears to be leaky (as also reported by

response to reviewers

others, see ref. 20), but pointing out that despite this leakiness and a 37% reduction in serum MSTN

levels, there were significant differences in body composition over time relative to each group's baseline

(lines 514-530) which is a major finding.
