# Supplementary material for: Sex differences in body composition but not neuromuscular function following long-term, doxycycline-induced reduction in circulating levels of myostatin in mice
Source: PLoS One. 2019 Nov 21;14(11):e0225283. doi: 10.1371/journal.pone.0225283 (PMC6872155; doi:10.1371/journal.pone.0225283)
Supplement: Attachment — Submitted filename: Response to reviewer(rev2).docx [file pone.0225283.s003.docx]

October 18, 2019

William D. Phillips

Academic Editor

PLOS ONE

Dear Editor,

On behalf of my collaborators, I wish to thank you for considering our manuscript and forwarding the reviewer’s critique. We have now address those comments as follows:

Reviewer Concern:

First, data has been added to Table S1, but there is an error in data presentation that needs correction. CMAP amplitude is stated as about 80mV, with SMUP at 232mV. It is not possible for these data to be correct and to lead to the stated MUNE. Almost certainly, the SMUP data should be in microvolts, or listed as ~0.2mv instead of ~200mV.

*Thank you for your attention to detail. You are correct that it is impossible to arrive at the reported MUNE values with the given information. It is SMUP that should be reported in microvolts. This has been changed in the Table S1.*

Reviewer Concern:

The authors acknowledge that the technique provides only an estimate of motor unit number, so our confidence in the accuracy of the estimate would be enhanced if the numbers were similar to estimates using other techniques, ie how does this result correlate with other methods that have assayed motor neuron (or motor unit) number to the triceps surae group in the mouse? So, my previous comment was asking the authors to reconcile this discrepancy between their TS motor unit number estimates (~350) and those of other authors (~150), acquired using other techniques. Note that I am not questioning or disputing the outcome showing a decrease in motor unit number with age, and I am not suggesting that any particular dataset is correct or incorrect, but (in my view) the discrepancy between the datasets is big enough to require comment.

*Author Response:*

*Thank you for the clarification of your concern. A common criticism of all MUNE techniques is that the absolute numbers of estimated MUs in a given muscle are discordant with anatomic counts, however, the relative values are consistent. There are several factors that make direct comparison between studies difficult, including differences in genetic backgrounds, age of the mice, and different techniques employed. The benefits of MUNE include the relatively short time required to perform the measure, ability to perform repeated measures in vivo, and reliability. Our group and others have consistently shown MUNE to be a reliable and sensitive biomarker of motor unit function [1-5]. We have added a statement in the limitations section addressing your concern (Page 20). A recent review on the utility and shortcomings of MUNE in clinical practice may shed additional light [6] but we have not included this citation because we feel it is beyond the scope of our present manuscript. Similarly, the advent of stereological techniques have manifested the shortcomings of earlier estimates of neuronal cell counts, which are being addressed with technological improvements [7]. However, this aspect is beyond the scope of our work.*

*1.	Sheth KA, Iyer CC, Wier CG, Crum AE, Bratasz A, Kolb SJ, et al. Muscle strength and size are associated with motor unit connectivity in aged mice. Neurobiol Aging. 2018;67:128–6.*

*2.	Arnold, W.D., P.N. Porensky, V.L. McGovern, C.C. Iyer, S. Duque, X. Li, et al. Electrophysiological Biomarkers in Spinal Muscular Atrophy: Preclinical Proof of Concept. Ann Clin Transl Neurol 2014; 1: p. 34-44.*

*3.	Arnold, W., V.L. McGovern, B. Sanchez, J. Li, K.M. Corlett, S.J. Kolb, et al. The neuromuscular impact of symptomatic SMN restoration in a mouse model of spinal muscular atrophy. Neurobiol Dis 2016; 87: p. 116-23.*

*4.	Kang, P.B., C.L. Gooch, M.P. McDermott, B.T. Darras, R.S. Finkel, M.L. Yang, et al. The motor neuron response to SMN1 deficiency in spinal muscular atrophy. Muscle Nerve 2014; 49: p. 636-44.*

*5.	Duque, S.I., W.D. Arnold, P. Odermatt, X. Li, P.N. Porensky, L. Schmelzer, et al. A large animal model of spinal muscular atrophy and correction of phenotype. Ann Neurol 2015; 77: p. 399-414.*

*6.	de Carvalho, M., Barkhaus, P.E., Nandedkar, S.D., and Swash, M., Motor unit number estimation (MUNE): Where are we now? Clin Neurophys 2018; 129: p. 1507-1516.*

*7.	Zygelyte, E., Bernard, M.E., Tomlinson, M.E., et al., RetroDISCO: Clearing technique to improve quantification of retrograde labeled motor neurons of intact mouse spinal cords. J Neurosci Meth 2016; 271: p. 34-42.*

Thank you very much for your consideration, and best regards,
